# Early Outcome Data Assessing Utility of a Post-Test Genomic Counseling Framework for the Scalable Delivery of Precision Health

**DOI:** 10.3390/jpm8030025

**Published:** 2018-07-25

**Authors:** Amy C. Sturm, Tara Schmidlen, Laura Scheinfeldt, Shelly Hovick, Joseph P. McElroy, Amanda E. Toland, J. Scott Roberts, Kevin Sweet

**Affiliations:** 1 Division of Human Genetics, Ohio State University Wexner Medical Center, Columbus, OH 43221, USA; asturm@geisinger.edu (A.C.S.); Amanda.Toland@osumc.edu (A.E.T.); 2 Genomic Medicine Institute, Geisinger, Danville, PA 17821, USA; tjschmidlen@geisinger.edu; 3 Coriell Institute for Medical Research, 403 Haddon Avenue, Camden, NJ 08103, USA; lscheinfeldt@coriell.org; 4 School of Communication, Ohio State University, Columbus, OH 43214, USA; hovick.1@osu.edu; 5 Department of Biomedical Informatics, Center for Biostatistics, The Ohio State University, Columbus, OH 43221, USA; Joseph.McElroy@osumc.edu; 6 Department of Health Behavior & Health Education, University of Michigan School of Public Health, Ann Arbor, MI 48109, USA; jscottr@umich.edu

**Keywords:** genetic, genomic, counseling, service delivery, risk awareness, recall, telegenetic, telephone, in-person

## Abstract

Information on patients’ preferences is essential to guide the development of more efficient genomic counseling service delivery models. We examined patient preferences in the context of use of a post-test genomic counseling framework on patients (*n* = 44) with chronic disease receiving online test reports for eight different diseases and one drug-response result. We also explored patients’ disease risk awareness, recall of test report information, and confidence in knowing what to do with their test results. Prior to the post-test genomic counseling session, all participants viewed at least one test report; 81.6% of available test reports were reviewed in total. Participants requested more phone (36) than in-person counseling sessions (8), and phone sessions were shorter (mean 29.1 min; range 12–75 min) than in-person sessions (mean 52.8 min; range 23–85 min). A total of 182 test reports were discussed over the course of 44 counseling sessions (mean 4.13, range 1–9). Thirty-six (81.8%) participants requested assessment for additional medical/family history concerns. In exploring patient experiences of disease risk awareness and recall, no significant differences were identified in comparison to those of participants (*n* = 199) that had received in-person post-test genomic counseling in a parent study randomized controlled trial (RCT). In summary, a novel post-test genomic counseling framework allowed for a tailored approach to counseling based on the participants’ predetermined choices.

## 1. Introduction

Novel approaches for effective genomic counseling are needed to meet increasing demand and to provide better efficiency. The continued integration of precision health into clinical practice will require re-evaluation of conventional approaches as more patients seek genomic counseling and testing. The conventional practice of in-person genetic counseling includes both pre-test and post-test components [1], an approach that is labor and cost-intensive [2] and impractical on a large scale [3,4]. Conventional practice is also provider driven, in that the counselors speak more than the clients [5], even though many clients may prefer to learn about their genetic risks in different ways, at different rates, and when it is convenient to them [6]. Use of alternative modes of service delivery to include genetic counseling such as by telephone or telegenetics (interactive video and a secure high-speed connection) [7,8,9,10,11] are well accepted by patients and decrease the amount of provider time [1,3,12]. These alternative modes are equivalent with respect to educating and supporting patients, attending to psychosocial issues, facilitating decision-making, and improving quality of life outcomes when compared to conventional genetic counseling [10,13]. Utilization of technology may also help facilitate patient access to services that are limited due to geographical or financial barriers [1]. Limitations to the use of telephone or telegenetics include the inability to fully assess non-verbal behaviors/cues, the need for additional support and/or access to technology (e.g., internet), the potential for dropped or interrupted communication, and the need for additional support for targeted patient populations (e.g., minority women or rural populations) [14].

As previously reported, we developed a novel post-test genomic counseling framework [15] based on qualitative research [16] and key health behavior theories [17]. Following receipt of genomic-based results, the counseling framework provides the patient with an opportunity to set the counseling agenda by selecting the specific test results they wish to discuss, specifying questions for discussion, and indicating their preference for communication modality (telephone, telegenetics or in-person). The counselor uses these patient preferences to tailor the genomic counseling session and to personalize result communication and risk reduction recommendations. Tailored visual aids and result summary reports divide areas of risk (genetic variant, family history, lifestyle) for each disease to facilitate the discussion of multiple disease risks and drug-response findings. Post-test genomic counseling session summary reports are actively routed through the electronic health record and patient facing portal to both the patient and their healthcare provider team to encourage review and follow-up (e.g., screening, preventive health behaviors).

In the demonstration project evaluated here, we focus on the application of this novel genomic counseling framework [15] on patients with chronic disease who have received online genomic-based test reports. This demonstration project had several aims. First, we examined participant preferences for: (1) communication modality (telephone, telegenetics or in-person genomic counseling), (2) the number of test reports viewed prior to counseling and requested for discussion by the participant, (3) the number of requests for counseling on additional personal medical/family history concerns beyond that included on the test reports, and (4) consultation time with the genetic counselor. Second, we explored whether use of this novel genomic counseling framework was associated with disease risk awareness, recall of test report information, and confidence in knowing what to do with test results. We compared these patient experiences to those of patients with chronic disease that had received in-person post-test genomic counseling in a parent study randomized controlled trial (RCT) [18].

## 2. Materials and Methods

**Figure 1:**
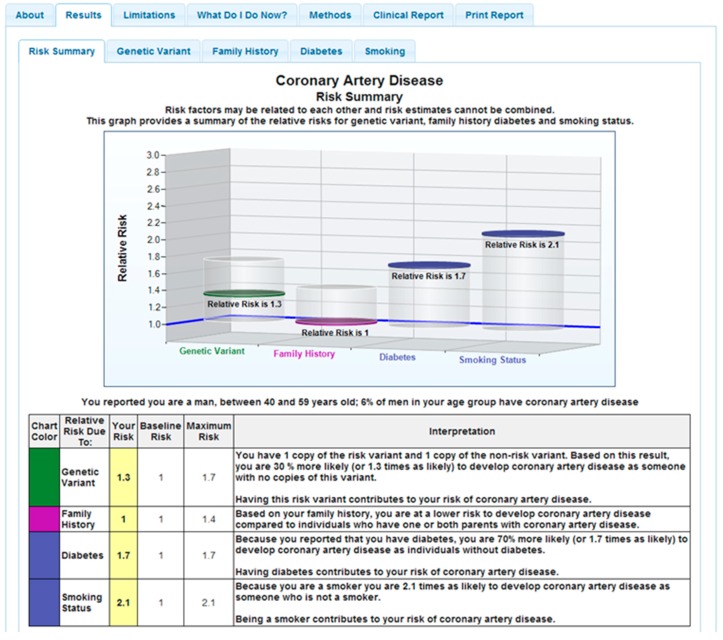
Sample CPMC (Coriell Personalized Medicine Collaborative) coronary artery disease report. Legend: Solid discs represent the participant’s relative risk and vertical cylinders depict the range of relative risk (RR) values possible for the risk variable. On-line risk reports are organized using a tabbed approach, with separate tabs for disease condition information, risk results, limitations, methods or review educational material. To ensure readability, the CPMC test report design was informed by multiple rounds of pilot testing conducted by allowing individuals with no scientific background to review report drafts and provide feedback.

Participants in the Coriell Personalized Medicine Collaborative (CPMC) received reports for 19 complex disease and seven drug-gene pairs through a secure web portal [19]. CPMC participants were administered surveys that collected demographic, medical and family histories, lifestyle, and medication information which were used to produce personalized risk reports. These reports present risk information as relative risk for each disease. Individual risk was based on genetic variant, family history, medical history, and health behavior risk factors and was presented in both graphical and numeric format (Figure 1). The pharmacogenomics reports provided information pertaining to predicted drug response and a corresponding interpretation. The CPMC web portal also provided text and multimedia educational materials and tools that enabled study participants to learn more about basic genetics concepts, complex disease genetics and concepts, pharmacogenomics, family history risk, relative risk and health condition-specific summaries detailing disease etiology, risk factors, treatment and available preventative or risk reducing actions. Results from primary outcomes of various trials related to the CPMC have been previously reported [16,18,20,21,22,23].

Study Design and Participants: Participants in this study were also part of a distinct ancillary study (The Ohio State University-Coriell Personalized Medicine Collaborative (OSU-CPMC) comprised of adult patients diagnosed with either hypertension or congestive heart failure. All OSU-CPMC participants received an initial batch of results pertaining to eight health conditions (coronary artery disease, type 1 diabetes, type 2 diabetes, hemochromatosis, melanoma, age-related macular degeneration, prostate cancer, and lupus) and 1 drug response report (*CYP2C19* and Clopidogrel) through an online portal. Once OSU-CPMC participants completed all the required study activities, they received additional disease risk and pharmacogenomics reports on a monthly basis until all available study reports were delivered online. Results from a RCT assessing in-person post-test genomic counseling on 199 OSU-CMPC participants were published previously [18]. The in-person sessions for the RCT counselees lasted between 60–90 min [18].

**Demonstration Project (DP):** To assess the utility of a pre-established genomic counseling framework [15], an additional cohort of 55 patients with chronic disease was recruited to the OSU-CPMC study. Institutional review board approval was obtained (#2014H0358), and patients were recruited between November 2014 and February 2015. Adult patients (*n* = 61) diagnosed with either congestive heart failure or hypertension were identified as study eligible by OSU Family Medicine physicians (*n* = 5). Eligible patients were contacted by a trained study recruiter to participate in one of three offered group education/consent sessions. In these sessions, they were administered a PowerPoint educational presentation that covered background information on DNA, genes, and single nucleotide polymorphisms (SNPs); the genetic basis of common, complex disease and pharmacogenomics; and logistics including access to the online OSU-CPMC web portal and the composition of the online test reports. Study recruiters also explained that from qualitative research conducted on OSU-CPMC study participants [16], investigators had developed and planned to test a new genomic counseling framework as part of a demonstration project (DP).

**Figure 2:**
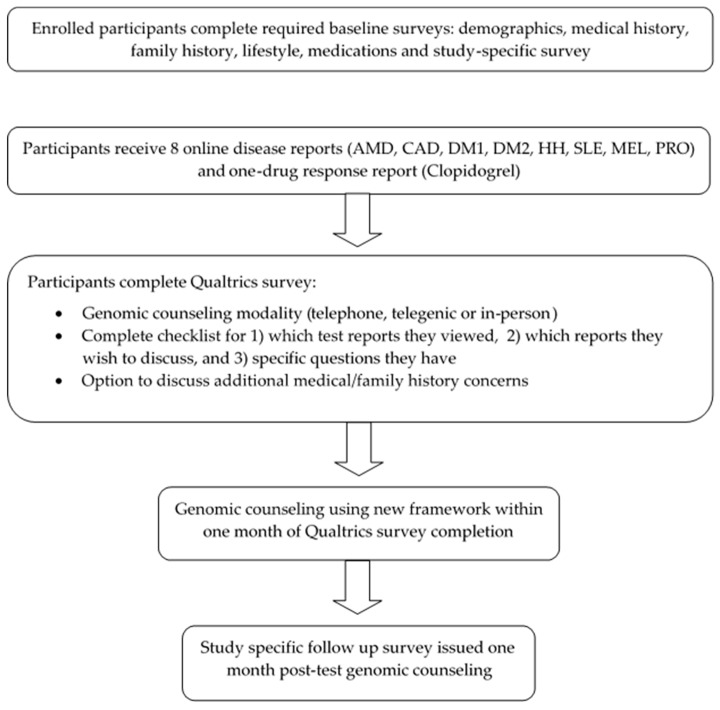
Study schematic. Legend: AMD: age-related macular degeneration; CAD: coronary artery disease; DM1: type 1 diabetes; DM2: type 2 diabetes; HH: hemochromatosis; SLE: systemic lupus erythematosus; MEL: melanoma; PRO: prostate cancer.

In all, 55 (90.1%) of 61 eligible patients were recruited to the DP and completed baseline surveys (Figure 2). DP participants then received email notice of the availability for online viewing of their nine initial test reports. Participants had the option to choose whether to view each test report. When viewing a test report, participants were initially directed to an OSU-CPMC webpage containing written and video-based educational material describing the specific condition, the role of each risk factor, and approaches to prevention and treatment. Participants could also choose not to view these educational materials and proceed directly to their individual test reports.

Demonstration project participants subsequently received an email notification reminding them of the availability of free genomic counseling. This email provided a secure link to a Qualtrics survey, which was designed to elicit participant preferences for the post-test genomic counseling session (Supplementary Figure S1). The survey provided: (1) options for counseling modality (telephone, telegenetics or in-person); (2) a checklist for which of nine test reports the participant had viewed, which reports they would like to discuss, and the option to ask specific questions; and (3) an option to list additional medical/family history concerns beyond those associated with the test reports. Completion of the Qualtrics survey allowed investigators to reach out by phone/email to schedule the genomic counseling appointment.

One of two licensed genetic counselors provided genomic counseling to DP participants. These were the same genetic counselors (A.C.S., K.S.) that provided in-person counseling for the RCT [18]. Specifically, the genetic counselor utilized the participant preferences from the Qualtrics survey to tailor the genomic counseling session, personalize result communication, and risk reduction recommendations following a semi-scripted template (Supplementary Figure S2). To facilitate learning and discussion of multiple disease risks, the genomic counseling framework utilized personalized visual aids and result summary reports to divide and highlight areas of risk (genetic variant, family history, behavior/lifestyle) for each disease [15]. For phone counselees, these documents were secure-emailed 24 h prior to the genomic counseling session. In addition to discussing any test reports requested by the participant, if there was any increased risk (genetic variant, family history, behavior/lifestyle) noted in a test report, the genetic counselor discussed these risks. If the counselee had asked on the Qualtrics survey for assessment of additional personal medical/family history concerns beyond that afforded by the nine test reports, this was provided. After the post-test genomic counseling session, summary reports were actively routed to the DP participant by secure email, and their healthcare provider team through the electronic medical record (EMR), to encourage additional review and follow-up.

### 2.1. Patient Preferences, Patient Experience Measures and Statistical Analyses

#### 2.1.1. Patient Preferences

Patient preferences were assessed by reviewing: (1) choice for communication modality (telephone, telegenetics or in-person counseling); (2) the number of test reports viewed, and requested for discussion; (3) the number of additional questions on personal medical/family history concerns beyond those associated with the test reports; and (4) consultation time with the genetic counselor.

#### 2.1.2. Patient Experience Measures

Demonstration project participants completed pre- and post-test genomic counseling surveys to measure patient experiences, including recall and accuracy of their actual disease risk identified in the test report(s) they had requested for discussion. Additionally, we evaluated personal (1) disease risk awareness, (2) risk accuracy and recall of test report information, and (3) perceived confidence in knowing what to do with test results, and compared these patient experiences to those of RCT counselees that had received in-person genomic counseling and had completed the same patient experience measures [18].

Accuracy of actual risk for test reports requested for discussion: To assess whether or not a specific test report requested for discussion by the participant influenced accurate understanding of their risk, we first reviewed the number of test reports requested and then assessed risk accuracy by comparing their response on the follow-up survey for each disease (“Do you have an increased risk for any of the following conditions due to family history, genetic variant, non-genetic risk factors?”). For this binary variable (discussed vs. not discussed), we required at least 10 participants per group to perform analyses. For each disease, the follow-up risk answer was modeled with ordinal logistic regression with co-variates of gender, age, and education, and a main effect of discussed/not-discussed. Baseline data were not available for these questions. Logistic regression was used to model follow-up accuracy of risk including the co-variates baseline correctness, gender, age, education, and a main effect for discussed/not-discussed. The latter analyses were performed within risk type (variant, family history, or lifestyle/behavior). Comparison-wise raw *p* = 0.05 was considered the significance threshold herein.

### 2.2. RCT Comparisons

#### 2.2.1. Disease risk awareness

A participant’s causal attribution of risk for each disease was assessed for each risk factor at baseline and follow-up (e.g., “How much do you think having a genetic risk variant determines whether or not a person will develop each of the following conditions?”) [24]. Five point Likert scales were used and ratings were combined across all eight diseases (e.g., macular degeneration) to generate composite scores of the overall importance a participant placed on genetic variants, family history, and lifestyle/behavior for disease risk. Cronbach’s alphas were >0.80 at baseline, respectively, for these composite items. A linear model was fit to the composite follow-up scores with co-variates for baseline composite score, gender, age, education level, and a main effect for group.

We assessed each participant’s personal awareness of risk due to family history (“Do you have an increased risk for any of the following conditions due to your family history?”) and lifestyle/behavior factors (“Do you have an increased risk for any of the following conditions due to lifestyle/behavior factors (for example, smoking, poor diet, high body mass index (BMI)?”) at baseline and at follow-up for each disease. Personal awareness of risk based on the addition of genetic variant risk for each disease was then also assessed only at follow-up (“Do you have an increased risk for any of the following conditions due to a genetic risk variant?”). Response options for these questions included: yes, no, not applicable, don’t know, do not want to answer. We compared these responses to the actual risk for each disease based on what was indicated in the test report (Supplementary Figure S1). A logistic regression model was employed to test the accuracy of risk (correct/incorrect) within each disease and risk type (lifestyle/behavior/family history), with co-variates for baseline correctness, gender, age, education level, and a main effect for group. As baseline data for the genetic variant did not exist for this dataset, it was not included as a co-variable in the analyses. To compare confidence in personal awareness of risk (know vs. do not know if at increased risk, for the genetic variant only), a 2 × 2 Fisher’s exact test (FET) was employed for comparison between DP participants and RCT counselees.

#### 2.2.2. Perceived Risk and Recall of Risk

To assess each participant’s perceived risk of developing a particular disease, and their recall of this risk, we used a single question at follow-up only (“What do you think is your chance of developing each of the following diseases in your lifetime?” [25]). Using a 5-point Likert scale (1, certain not to happen; 5, certain to happen) for each disease, the participant’s answer was modeled with ordinal logistic regression with co-variates of gender, age, and education, and a main effect of group.

#### 2.2.3. Confidence in knowing what to do with test results

We assessed confidence in use of multiple test results with their level of agreement with the statement: “I know what to do with my results”. To compare confidence in use of these results, the follow-up answer (1–5) was modeled with ordinal logistic regression with co-variates of gender, age, and education, and a main effect of group.

## 3. Results

Socio-demographics: Table 1 depicts enrollment numbers and socio-demographic information. Of 55 DP participants enrolled, two were subsequently removed from study because they failed to complete required baseline surveys. All 53 DP participants received an initial batch of nine test reports, with 44 (83%) subsequently completing the pre-session Qualtrics survey. Forty-two participants (95.5%) completed the study follow up surveys.

Patient Preferences: Of 44 DP participants, 36 (81.8%) chose to have telephone counseling (mean, 29.1 min; range 12–75 min); the remainder (18.2%) chose in-person counseling (mean, 52.8 min; range 23–85 min). No participants chose the telegenetics option. In comparison, as noted for RCT counselees, the conventional in-person counseling sessions lasted between 60–90 min. Based on analytic data obtained from the the web portal before post-test genomic counseling, all 44 DP participants viewed at least one test report, with 81.6% of the available online test reports reviewed in total; this was slightly more than RCT counselees (75.4%; Table 2). 

In all, 22 (50%) DP participants requested discussion of one or more test reports (total number of report requests, 75; mean, 1.70; range 0–9; Table 3). There were a total of 182 test reports discussed over the course of the 44 post-test genomic counseling sessions (mean 4.13, range 1–9). To assess any differences between DP and RCT participants and the viewing of test reports, we found a significantly higher percentage of DP participants viewed test reports for two diseases (Type 2 diabetes *p* = 0.019; melanoma, *p* = 0.047) but did not find an association between the number of test reports viewed and the number of topics discussed in the post-test genomic counseling session (*p* = 0.928). We also assessed the number of participant requests pre-session, for additional questions on personal medical/family history concerns beyond those associated with the test reports. Thirty-six (81.8%) DP participants requested additional topics for discussion in the post-test counseling session. For 18, there were concerns regarding both cancer and heart disease risks; 11 for cancer and three heart disease risk only; and three were “not sure”. Besides these specific concerns, there were 12 additional disease topics discussed in the post-test genomic counseling sessions (Table 3).

### 3.1. Patient Experience Measures

Accuracy of risk for test reports requested for discussion: For participants who asked to discuss a particular disease test report before counseling, we sought to determine if genomic counseling altered their personal awareness of risk for these diseases compared to those who did not ask to discuss a particular disease. We found that across risk type analyses, there were suggestive associations for two diseases (Table 4a: AMD (age-related macular degeneration), *p* = 0.166; SLE (systemic lupus erythematosus), *p* = 0.082). That is, there was a trend for participants that felt they were at a higher risk for AMD or SLE to ask to discuss these diseases in counseling. We evaluated the accuracy of their risk perceptions for family history, genetic variant and non-genetic risk for any diseases the participant asked to discuss in the post-test genomic counseling session. We found significant associations for the DM2 (type 2 diabetes) genetic variant (FET *p* = 0.035); that is, if DM2 was discussed, the participant’s accuracy of their perceived DM2 risk due to the genetic variant tended to be correct. Notably, for the SLE genetic variant, the association was in the opposite direction than expected (*p* = 0.036), in that if it was discussed, the participant was less often correct (Table 4b). Given that the relative risk afforded to the genetic variant for DM2 and SLE was quite similar (RR 1.2–1.3), while the relative risk for family history was more significant and complex for SLE (RR 4.0/11.0) than for DM2 (RR 1.3), we looked at the individual pedigrees, and assessment of family history risk for both diseases, pre- and post-test genomic counseling. We found that for the 13 participants who were accurate in their recall of SLE genetic variant risk, six did not provide a family history of SLE or other autoimmune disease upon entering the study; thus, family history risk was not included in their SLE test report. In the counseling session, more in-depth discussion of SLE family history risk was performed for these 13 participants, and family history risk was subsequently modified for five participants. We compared this assessment to the seven participants who were “incorrect” for recall of their SLE variant, and the five participants who were “incorrect” for recall of their DM2 genetic variant; for these 12 participants, disease risk afforded by family history was not modified by counseling. This suggests that participant’s recall of their SLE genetic variant risk may have been complicated by the family history risk, especially as history of 11 separate autoimmune diseases (e.g., lupus, rheumatoid arthritis, Sjogren’s, vitiligo, multiple sclerosis, celiac disease, type 1 diabetes, autoimmune hyperthyroidism aka Grave’s disease, Crohn’s disease, ulcerative colitis and psoriasis) are considered within the SLE family history risk assessment.

### 3.2. RCT Comparisons

In exploring patient experiences of disease risk awareness and recall, no significant differences (*p* > 0.05) were identified in comparison to RCT counselees. The majority of DP participants (92.3%) receiving post-test genomic counseling with the new framework expressed confidence (i.e., agree, strongly agree) in knowing what to do with their test results, which was comparable to that of RCT counselees who received in-person genomic counseling (*p* > 0.05; Table 5).

## 4. Discussion

Information on patients’ preferences, risk perceptions, and informational needs are essential to the provision of personalized, yet efficient, genomic counseling. In this demonstration project, we examined patient preferences in the context of use of a novel post-test genomic counseling framework on patients with chronic disease receiving multiple online genomic-based test reports. As a result of assessing patient preferences (communication modality preference, reports viewed/requested for discussion, requests for additional medical concerns, and consultation time), we found the new framework in practice to be more patient-driven and more flexible than conventional in-person approaches. The post-test genomic counseling framework allowed for a tailored approach to counseling based on the participants’ predetermined choices. Participants read more test reports and their risk awareness, recall of test results and confidence in knowing what to do with their test results was comparable to that of RCT participants. 

Our post-test genomic counseling framework was designed with a participant-driven focus in mind, with an additional intent to increase efficiency. This includes the use of online contracting (establishing the counseling agenda) pre-session with question prompts. This approach allowed the counselor to highlight and focus on what the patient wanted to discuss, versus a comprehensive review of all test results (even those for which there was no increased risk). This approach also allowed for opportunity to provide counseling and clarification of risks, as well as assess and discuss additional disease risk influences per the patients’ requests. We did not complete full 3–4 generation pedigrees during the session unless it became necessary to answer specific questions posed by the counselee. We did have baseline family history information provided pre-session by the participant through use of the OSU-CPMC web portal, and participants also had the opportunity to visit an online cancer and heart disease family history risk assessment tool [26]. This approach resulted in focused and shorter counseling sessions. The new post-test genomic counseling framework also allows for inclusion and discussion of multiple disease risk and pharmacogenomics results, as well as assessment of additional personal and family history risk concerns per the patient’s request. Much of the extra discussion centered on personal or family cancer or heart disease risks, which at times bridged into Mendelian disease risks, illustrating how this approach allows for counseling for both rare and common disease risk influences, and applicability for any type of genetic/genomic result.

A number of studies (e.g., OSU-CPMC; My46; PGen) have now explored the provision of genomic results through interactive web-based information systems for use in the research and clinical settings [19,27,28,29,30]. In general, these models of service delivery allow convenient, adaptable and scalable access to results in a confidential and secure manner. Furthermore, it allows patients or research participants to set and modify result return preferences, process results in their own time, and access self-directed education and learning tools [19,27,28,29]. Because of the numerous and varied genomic variants provided with different levels of risk (e.g., few are high risk, many are moderate or low risk) [31,32], allowing patients to access their results prior to genomic counseling gives them the opportunity to evaluate their results, formulate questions, and identify areas of interest or concern for discussion with their genetic counselor. Information technologies (e.g., laptops, hand held devices), and innovative health communication approaches, such as including increased access to on-demand information and communication with health professionals (e.g., phone, text, email or chat), engaging in social networking, and tailoring of information to the specific needs or characteristics of individuals or groups of users can also help facilitate delivery of genomic results [33]. Use of e-technologies could also provide an opportunity for education and support in a more participatory and less healthcare provider time-intensive fashion [33,34,35].

An increase in usage of conventional genetic testing and direct-to-consumer (DTC) services has created tremendous demand and genetic counselors struggle to be more efficient without sacrificing quality of care. Importantly, between 2016 and 2018, almost half of practicing genetic counselors reported an increase in patient volume [36]. The number of genetic counselors available to help patients parse through genetic test results, likewise, is not keeping pace with the demand [36,37]. Currently there are only about 4000 certified genetic counselors in the U.S. [36]. The most common model of service delivery remains in-person, although in 2018, 62% of genetic counselors reported using more than one delivery model (e.g., telephone; telegenetics) to interact with their patients [36]. The post-test genomic counseling framework was designed to be more flexible for use in a variety of genomic counseling settings and alternative methods of service delivery. The framework could also be further developed and expanded upon to incorporate any type of potentially actionable genomic test result (e.g., exome sequencing), to include rare Mendelian in addition to common multifactorial disease (e.g., polygenic risk scores). An effective post-test genomic counseling framework in the clinical setting would have significant public health implications for modifying conventional genetic counseling, with the potential to increase efficiency and ultimately reduce costs and resource limitations.

Further integration of genetic and genomic counseling services within the genomic results delivery process is essential [38,39]. Development of service delivery frameworks that are more participant-driven is also timely given the rise of large, population-wide efforts of genomic sequencing to include the National Institutes of Health All of US^SM^ Research Program [38], the Geisinger MyCode Community Health Initiative, and other initiatives [40,41]. The ultimate goal is to provide more accurate predictions of risk for multiple diseases and medical indications, while utilizing and expanding upon online and interactive service delivery platforms so that individuals can take a more personalized, preventive and participatory approach to their health [38].

**Limitations:** The between study comparisons are confounded by known and potentially unknown variables such as differences in distribution of disease diagnoses as well as limited sample size. Our participants were for the most part Caucasian and well educated. Some data was self-reported (e.g., family history). Use of only two genetic counselors for the post-test genomic counseling sessions and the use of a study-specific web portal may reduce the generalizability of study findings.

## Figures and Tables

**Table 1:** Demographic information.

Demographic Category	Subject Category	DP (*n* = 53)	RCT (*n* = 75)	*p*-Value	Test
Mean Age (sd)		58.89 (10.55)	57.73 (13.58)	0.59	*t*-test
Education (mode (%))		Graduate Degree (47%)	Graduate Degree (33%)	0.273	Fisher’s Exact Test
Race (Caucasian)	Yes	47	67	1	Fisher’s Exact Test
	No	6	8		
Gender	Female	31	32	0.106	Fisher’s Exact Test
	Male	22	43		
Income	<$25k	2	5	0.3406	Fisher’s Exact Test
	$25–50k	4	12		
	$50–75k	15	19		
	$75–100k	11	19		
	>$100k	21	19		
	Did not want to answer	0	2		
Diagnosis	HTN	51	42	<0.05	Fisher’s Exact Test
	HF	2	33		
Heath Care Occupation	Yes	8	9	0.609	Fisher’s Exact Test
	No	45	66		

Legend: DP: demonstration project participants; HTN: hypertension; HF: heart failure; RCT: randomized controlled trial counselees; sd: standard deviation.

**Table 2:** Number of test risk reports viewed post-test genomic counseling.

Disease	DP (*n* = 44)	RCT (*n* = 75)
AMD	38 (86.4%)	70 (93.4%)
CAD	37 (84.1%)	63 (84.2%)
DM1	37 (84.1%)	53 (71.1%)
DM2	40 (91.0%)	54 (72.4%)
HH	34 (77.3%)	58 (77.6%)
SLE	36 (81.8%)	53 (71.1%)
MEL	38 (86.4%)	52 (69.7%)
PRO	30 (68.2%)	44 (59.2%)
Plavix	33 (75%)	53 (71.1%)
**TOTAL**	**323 (81.6%)**	**509 (75.4%)**

**Table 3:** Number of test reports and additional concerns requested for discussion.

Participant	Number of Test Reports Viewed Pre-Session by Participant	Number of Test Reports Requested for Discussion	Test Reports Discussed in Post-Test Counseling	Cancer and/or Heart Disease Concerns	Additional Specific Concerns
1	All	DM1, SLE	DM1; DM2; SLE	Cancer	-
2	AMD, DM1, DM2, CAD, HH, MEL, PRO, SLE	AMD, CAD, DM2, SLE	AMD, CAD, DM2, SLE	Both	-
3	All	None checked	DM2	Both	Family history Lynch syndrome
4	All	None checked	AMD, CAD, DM2, MEL, SLE	Cancer	Family history antiphospholipid antibody syndrome
5	AMD, DM1, DM2, CAD, MEL, PRO, SLE, Plavix	None checked	AMD, CAD, SLE	-	-
6	AMD, DM1, DM2, CAD, MEL, PRO, SLE, Plavix	None checked	AMD, CAD, DM2, SLE	-	-
7	AMD, DM2, CAD	CAD	CAD, DM2, SLE	Both	BRCA1 mutation carrier
8	AMD, DM1, DM2, CAD, HH, MEL, SLE	AMD, DM1, DM2, CAD, HH, MEL, SLE, Plavix	AMD, DM1, DM2, CAD, HH, MEL, SLE, Plavix	Both	-
9	All	None checked	AMD, DM2, SLE	Cancer	-
10	All	AMD, DM2, CAD, MEL	AMD, DM2, CAD, MEL, SLE	Both	-
11	AMD, DM1, DM2, CAD, HH, MEL, SLE, Plavix	None checked	CAD, DM2, MEL	-	-
12	All	AMD, DM1, DM2, SLE	AMD, DM1, DM2, MEL, SLE	Both	-
13	All	AMD, DM1, DM2, CAD, MEL, SLE	AMD, DM1, DM2, CAD, MEL, SLE	Both	-
14	All	None checked	AMD, CAD, DM2, SLE	Both	-
15	DM1, DM2, HH, MEL, SLE	None checked	AMD, CAD, DM2, SLE	Both	Family history thrombophilia
16	AMD, SLE, Plavix	AMD, SLE, Plavix	AMD, DM2, MEL, SLE, Plavix	Not sure	Family history congestive heart failure
17	All	None checked	AMD, CAD, DM2, SLE	Heart disease	-
18	All	DM1, DM2, CAD, MEL	AMD, CAD, DM1, DM2, MEL, SLE	Both	Personal history of colon polyps
19	AMD, DM1, DM2, CAD, HH, MEL, PRO, SLE	Plavix	CAD, DM2, PRO, SLE, Plavix	Cancer	-
20	All	PRO; Plavix	CAD, DM2, PRO, SLE, Plavix	Cancer	-
21	All	AMD, DM1, DM2, CAD, HH, MEL, PRO, SLE, Plavix	AMD, DM1, DM2, CAD, HH, MEL, PRO, SLE, Plavix	Not sure	Personal history of cholesterol and hypertension
22	All	CAD	AMD, CAD, DM2	Heart disease	-
23	CAD	AMD, PRO	AMD, CAD, DM2, PRO	-	-
24	AMD, DM1, DM2, CAD, MEL, PRO SLE, Plavix	DM1, DM2	DM1, DM2	-	Personal history of cholesterol and hypertension
25	DM2	None checked	AMD, CAD, DM2, SLE	Heart disease	-
26	All	None checked	DM2, SLE PRO	Both	Family history Tetralogy of Fallot
27	AMD, DM1, DM2, CAD, HH, MEL, SLE, Plavix	DM2, CAD, SLE	CAD, DM2, MEL, SLE	Both	Family history intestinal malrotation
28	AMD, DM1, DM2, CAD	None checked	CAD, DM2	-	-
29	DM2	None checked	AMD, CAD, DM2, MEL, SLE	Cancer	-
30	All	CAD, Plavix	AMD, CAD, DM2, Plavix	-	-
31	All	None checked	AMD, DM2, PRO	Cancer	-
32	CAD, DM1, DM2, HH, MEL, Plavix	DM1, CAD, Plavix	CAD, DM1, DM2, SLE, Plavix	Both	Family history kidney failure
33	AMD, CAD, MEL, PRO, Plavix	None checked	AMD, CAD	Both	Family history depression
34	All	AMD, CAD, MEL, PRO, Plavix	AMD, CAD, DM2, MEL, PRO, SLE, Plavix	Both	-
35	AMD, DM2, CAD, HH, MEL, PRO, SLE, Plavix	None checked	AMD, CAD, DM2	Cancer	-
36	All	None checked	AMD, CAD, DM2	Cancer	-
37	All	AMD, DM2, MEL, PRO	AMD, CAD, DM2, MEL, PRO, SLE	Both	-
38	All	None checked	CAD, MEL, SLE	-	-
39	AMD, DM1, DM2, CAD, HH, MEL, SLE, Plavix	None checked	AMD, CAD, DM2, SLE	Both	-
40	All	AMD, CAD, PRO, SLE	AMD, CAD, DM2, PRO, SLE	Not sure	-
41	All	None checked	AMD, CAD, DM2, SLE	Cancer	-
42	DM2, MEL	Plavix	AMD, CAD, DM2, SLE, Plavix	Cancer	-
43	All	None checked	CAD, DM2, MEL, PRO, SLE	-	-
44	All	None checked	CAD, DM2	Both	-
**TOTAL**	**323**	**75**	**182**	**36**	**12**

**Table 4:** (**a**) Personal awareness of risk for any test reports the participant requested for discussion. (**b**) Accuracy of risk perception.

(**a**)				
**Disease**	**Estimate**	**Std Error**	***z*-Value**	***p*-Value**
AMD	−0.962	0.694	−1.387	0.166
SLE	−1.272	0.731	−1.74	0.082
(**b**)				
**Disease risk factor**	**Estimate**	**Std Error**	***z*-Value**	***p*-Value**
AMD variant	1.346	1.269	1.061	0.289
CAD family history	5.349	6.974	0.767	0.443
CAD variant	1.70	1.334	1.274	0.202
DM2 family history	−164.0	240	−0.0007	0.999
DM2 variant	1.863	0.885	2.105	0.035
SLE family history	−161.2	212	−0.0007	0.999
SLE variant	−3.359	1.598	−2.102	0.036

**Table 5:** Confidence in knowing what to do with test results.

	**SD**	**D**	**N**	**A**	**SA**	**DNWA**	**A%**	**D%**
DP	1	1	1	30	6	0	92.3%	0.05%
RCT	0	0	9	35	14	1	83.1%	0%
**DP Comparison to RCT**								
**Estimate**		**Std. Error**	***z*-Value**	***p*-Value**	**2.5% CI**	**97.5% CI**		
0.236		0.443	0.532	0.595	−0.634	1.114		

A: agree; CI: confidence interval; D: disagree; DNWA: did not want to answer; DP: demonstration project participants; N: neutral; RCT: RCT counselees; SA: strongly agree; SD: strongly disagree.

## Supplementary Materials

The following are available online at http://www.mdpi.com/2075-4426/8/3/25/s1. Figure S1: Qualtrics Survey; Figure S2: Post-Test Genomic Counseling Semi-Scripted Template.
