# Supplementary material for: Early Outcome Data Assessing Utility of a Post-Test Genomic Counseling Framework for the Scalable Delivery of Precision Health
Source: J Pers Med. 2018 Jul 25;8(3):25. doi: 10.3390/jpm8030025 (PMC6164140; doi:10.3390/jpm8030025)
Supplement: Supplementary file 1 [file jpm-08-00025-s001.zip › jpm-307487-supplementary 2.pdf]

Supplementary Figure SII: Post-Test Genomic Counseling Semi-Scripted Template

1. Contract
    a. Discuss the flow of counseling session initially driven by participants questions on Qualtrics survey
    b. Discuss the use of risk summary report and visual aids
2. Address disease risk(s) as noted by participant on Qualtrics survey
    a. Utilize test results summary report
    b. Discuss and assess medical/family history relative to a particular disease and/or test report
    c. Use visual aids to illustrate magnitude of risk
    d. Access web portal to co-view test reports, other educational aids with participant if needed
3. Discuss and assess additional personal medical/family history concerns
4. Psychosocial assessment
5. Develop action plan
6. Inform participant that the risk summary reports and a summary letter will be actively routed to the participant by secure email, and their healthcare provider team through the EMR, to encourage additional review and follow-up
