# Supplementary material for: Early Outcome Data Assessing Utility of a Post-Test Genomic Counseling Framework for the Scalable Delivery of Precision Health
Source: J Pers Med. 2018 Jul 25;8(3):25. doi: 10.3390/jpm8030025 (PMC6164140; doi:10.3390/jpm8030025)
Supplement: Supplementary file 1 [file jpm-08-00025-s001.zip › jpm-307487-supplementary.pdf]

Figure SI: Qualtrics Survey

This short survey asks you your preferences for genetic counseling. As part of the study you can speak or meet with a genetic counselor about your test results or any other questions you may have. This genetic counseling session is free (no charge). Once you provide your preferences, a member of our research team will contact you to schedule the session.

You have several options for genetic counseling. You can speak to the genetic counselor on the phone, meet with them in-person on the main OSU campus, or do the counseling session via online video conference. What is your preference?

- Phone
- In-Person
- Online Video Conference via WebEx
- Don't Care

Do you have a computer, tablet, or cell phone with internet access that you can use for an online video conference?

- Yes
- No

Please select your preferred format for the genetic counseling session.

- Phone
- In-Person
- Don't Care

Have you reviewed at least one of the nine Coriell test reports through online web portal? These test reports provided specific disease risk information based on genetic testing and other information you provided.

- Yes
- No
- Not Sure

Which of the following nine Coriell test reports have you looked at? Check all that apply.

- Type 1 Diabetes
- Type 2 Diabetes
- Hemochromatosis
- Lupus (SLE)
- Coronary Artery Disease
- Prostate Cancer
- Skin Melanoma
- Age-Related Macular Degeneration
- Plavix (Clopidogrel)
- I have not looked at the reports.

If you would like to discuss specific test reports with your genetic counselor, please click on that test report and write your specific questions in the space below.

- Type 1 Diabetes ____________
- Type 2 Diabetes ____________
- Hemochromatosis ____________
- Lupus (SLE) ____________
- Coronary Artery Disease ____________
- Prostate Cancer ____________
- Skin Melanoma ____________
- Age-Related Macular Degeneration ____________
- Plavix (Clopidogrel) ____________
- I have no questions about the reports.

Beyond the Coriell test reports, is there something else in your personal medical history or family history that you would like to discuss with the genetic counselor?

- Yes
- No
- Not Sure

Is what you would like to discuss related to any of the following diseases?

- Cancer
- Heart Disease
- Both Cancer and Heart Disease
- Neither Disease

What else in your personal medical history or family history would you like to discuss with the genetic counselor? ____________

Thank you for taking the time to fill out this short survey. We will reach out to schedule an appointment as soon as possible. In the meantime, you can access more information about the diseases and drug responses in the Coriell reports from the website below.

We thank you for your time spent taking this survey. Your response has been recorded.
